# Weather-Enhanced Machine Learning for Time-Resolved Risk Stratification of Clinically Managed Hymenoptera-Related Sting Events in an Urban German Region

**DOI:** 10.3390/ijerph23070881

**Published:** 2026-07-08

**Authors:** Mohamad Amer Nashtar, Theodor Baars, Nicoleta-Alexandra Stille, Isabella Traut, Ali Canbay, Klaus Zeppenfeld, Mustafa K. Özçürümez, Antonios Katsounas

**Affiliations:** 1 Ruhr University Bochum, Knappschaft Kliniken University Hospital Bochum, Department of Medicine, 44892 Bochum, Germany; mohamad.nashtar@rub.de (M.A.N.); theodor.baars@t-online.de (T.B.); isabella.traut@rub.de (I.T.); ali.canbay@rub.de (A.C.); mustafa.porsch-oezcueruemez@rub.de (M.K.Ö.); 2 Department of Computer Science, Campus Emil-Figge-Strasse, Dortmund University of Applied Sciences and Arts, 44227 Dortmund, Germany; nicoleta.stille@gmail.com (N.-A.S.); klaus.zeppenfeld@fh-dortmund.de (K.Z.)

**Keywords:** insect sting exposure, hymenoptera venom allergy, artificial intelligence, machine learning, weather-informed risk stratification, time-resolved risk assessment, digital health, environmental health

## Abstract

**Highlights:**

**Public health relevance—How does this work relate to a public health issue?**

- Insect stings are a relevant environmental and public health exposure, as they may trigger severe allergic reactions and anaphylaxis in susceptible individuals.
- This study links long-term clinically treated Hymenoptera-related sting events with high-resolution meteorological data to explore short-term, weather-informed exposure risk patterns.

**Public health significance—Why is this work of significance to public health?**

- The manuscript provides a proof-of-concept framework for linking routinely available weather data with clinical sting-event records to support exploratory, time-resolved risk stratification.
- By identifying intra-seasonal clustering and low-risk periods, the approach may contribute to future preventive risk-awareness strategies, particularly for individuals at increased risk of venom-induced systemic reactions.

**Public health implications—What are the key implications or messages for practitioners, policy makers and/or researchers in public health?**

- Weather-informed digital risk-awareness tools may complement established preventive measures but should not be used as stand-alone clinical warning systems without further validation.
- Future research should validate this approach in larger, multi-center cohorts and integrate behavioral, ecological, and exposure-related data before considering clinical, public health, digital health, or policy implementation.

**Abstract:**

Background: Insect stings, particularly those caused by Hymenoptera such as wasps and bees, are frequent triggers of severe allergic reactions and anaphylaxis, yet the ability to predict short-term risk periods based on environmental conditions has not been systematically evaluated. Meteorological factors influence both insect activity and human exposure, highlighting a relevant gap in preventive risk assessment. Methods: This exploratory single-center study was conducted in Bochum, Germany, an urban region within the Rhine-Ruhr metropolitan area. A 17-year retrospective dataset (2005–2022) of clinically treated Hymenoptera-related sting events was analyzed to explore time-resolved, weather-informed patterns using artificial intelligence (AI)-based machine learning. The study emphasizes methodological feasibility and pattern identification rather than clinical prediction. Daily weather parameters were transformed into expert-informed indicators capturing current-season and carry-over environmental conditions. A multilayer perceptron (MLP) was trained to identify periods of increased sting occurrence, and model performance was evaluated primarily using recall to capture rare-event signals. Results: A total of 346 clinically significant sting events were recorded. Weather variables showed strong spatial coherence across four stations and were associated with intra-seasonal clustering of sting events rather than absolute annual incidence. Exploratory analyses suggested that earlier seasonal onset correlated with higher sting counts (Pearson R = −0.52; *p* = 0.037). Weekly aggregation improved model performance compared with daily prediction. The cross-validated MLP showed moderate recall (0.431) and high specificity (0.86), supporting exploratory risk stratification; however, post hoc benchmarking did not demonstrate consistent superiority over simpler baseline approaches. Conclusions: This study combines a long-term clinical insect sting dataset with high-resolution meteorological data to explore time-resolved, weather-informed risk patterns using machine learning. The findings demonstrate the technical feasibility of exposure-based risk stratification in a rare-event setting. However, benchmarking showed that the MLP did not consistently outperform simpler baseline approaches for binary warning of elevated-risk periods. This proof-of-concept should therefore be interpreted as exploratory and not as a stand-alone warning system, supporting further external validation in larger, multi-center cohorts before clinical, public health, or digital health implementation can be considered.

## 1. Introduction

Insects, particularly Hymenoptera such as wasps and bees, play important roles within ecological systems by providing essential ecosystem services, but they may also pose health risks to humans through venomous stings [1,2]. From a clinical and public health perspective, Hymenoptera stings represent a relevant environmental exposure event because they may trigger reactions ranging from local symptoms to systemic allergic reactions and, in susceptible individuals, life-threatening anaphylaxis [3]. In Germany, sensitization to insect venom has been reported in approximately 25% of the population, whereas clinically relevant IgE-mediated systemic reactions after stings are considerably less common, with an estimated prevalence of approximately 3.5% and about 20 fatalities per year [4]. These figures indicate a relevant susceptible population; however, venom sensitization should not be equated with clinical allergy or anaphylaxis, as systemic reactions occur only in a subset of sensitized individuals following sting exposure.

The likelihood of systemic allergic reactions is influenced by individual sensitization status, previous sting reactions, and repeated venom exposure. Severe reactions occur in approximately 1% of children and up to 3% of adults [5]. Although venom sensitivity decreases significantly after childhood exposure, long-term follow-up data indicate that approximately one in five individuals may still develop an allergic reaction upon re-exposure more than 30 years later [5,6]. Higher reaction rates have been reported in highly exposed groups, such as beekeepers and their families, in whom frequent bee contact and repeated stings are associated with local allergic reactions in up to 31% and systemic anaphylaxis in 14–32% of individuals [5]. These estimates should therefore be interpreted in the context of sensitized or highly exposed populations rather than generalized to the entire population. Overall, sting exposure is a necessary prerequisite for venom-induced allergic reactions, whereas the occurrence and severity of systemic reactions depend on individual susceptibility, sensitization status, and exposure context. Therefore, reducing exposure to Hymenoptera stings remains an important preventive strategy, particularly for individuals with known or suspected venom allergies.

Importantly, the occurrence of a sting event is not equivalent to allergic disease or anaphylaxis, but it represents a necessary prerequisite for venom exposure [3]. At the population level, sting incidence therefore serves as a pragmatic and observable proxy for short-term exposure risk. Identifying periods with increased sting occurrence may support precautionary behavior and exposure avoidance, particularly in individuals at elevated risk of severe reactions.

Previous studies have primarily addressed the epidemiology of insect venom allergy and its clinical management, whereas data-driven approaches linking environmental conditions to short-term variation in sting occurrence remain limited [4,5,6,7]. Weather conditions are known to influence both insect activity and human outdoor behavior, suggesting that meteorological parameters may modulate temporal patterns of sting exposure [8]. However, no established framework currently exists to identify short-term, time-resolved periods of elevated sting risk from routinely available environmental data. This gap persists despite growing interest in artificial intelligence (AI)-based and digital approaches in allergology, which are increasingly explored for prediction, monitoring, and patient-centered management but still require context-specific validation [9,10,11].

To address this gap, an exploratory, proof-of-concept framework was developed combining long-term clinical sting data with meteorological information and AI-based machine learning to examine time-resolved patterns of clinically treated sting events. The outcome of interest was sting events requiring medical attention, rather than allergic sensitization or anaphylaxis itself. Although clinically relevant reactions may occur after exposure to wasps, bees, and other arthropods such as horseflies, these exposures were not modeled separately in the present analysis. The aim was not to predict absolute incidence, species-specific allergies, or direct clinical outcomes but to assess the technical feasibility of weather-informed, exposure-oriented risk stratification in a rare-event setting. Analogous to weather-integrated pollen forecasts for seasonal allergy sufferers [12], this approach may help identify periods of elevated environmental exposure. While primarily designed for civilian populations, it may also be relevant for groups with heightened outdoor exposure, including military personnel [13,14,15].

## 2. Materials and Methods

Predictive risk assessment modeling was performed using a structured four-step approach.

### 2.1. Clinical Insect Sting Data

This exploratory single-center retrospective study was conducted at Knappschaft Kliniken University Hospital Bochum. Bochum (population: 375,204) is an urban city within the Rhine-Ruhr metropolitan region, Germany’s largest urban agglomeration, with approximately 11.3 million inhabitants and a surface area of 7110 km^2^ [16]. This setting enabled linkage of long-term clinically treated sting events with regional meteorological observations from local and neighboring weather stations.

Step 1 involved the analysis of a retrospective clinical dataset of sting-related encounters covering the observation period from 31 July 2005 to 31 July 2022. Clinical sting events were identified retrospectively from the institutional hospital information system, including emergency department and inpatient clinical documentation, discharge diagnoses, and coding records. Case identification was based on the ICD-10-GM code T63.4 (“toxic effect of venom of other arthropods”) and was complemented by a review of free-text clinical annotations to confirm sting-related presentations [17]. The analysis therefore captured clinically treated sting events rather than systematically observed insect populations.

Variables were selected a priori based on their relevance for exposure-oriented risk stratification, availability across the full observation period, and consistent linkage with daily or weekly meteorological data. Core clinical variables included the date of the sting-related encounter, the presence of a clinically treated sting event, and species attribution where explicitly documented. Temporal variables, including year, month, calendar week, and season, were derived from the encounter date and linked to meteorological parameters described in Section 2.2 and Section 2.3.

The clinical dataset was accessed for research purposes between 1 March 2024 and 31 August 2025. Data were extracted retrospectively from the institutional hospital information system. The authors had no access to directly identifying information at any stage of data handling. All data were fully anonymized prior to analysis, and re-identification was not possible.

Species information was retained only when explicitly documented in the medical record, primarily distinguishing between wasp and bee stings. Cases without documented species attribution were classified as unknown and were not assigned to a specific insect species. Because species attribution was incomplete, the analysis focused on aggregated clinically treated Hymenoptera-related sting events rather than species-specific prediction. The ecological indicator framework was primarily informed by the seasonal biology of the German wasp (*Vespula germanica*), a medically relevant Hymenoptera species in Central Europe [18,19,20,21]. Documented wasp and bee sting events, including cases attributed to *Vespula germanica* and the Western honey bee (*Apis mellifera*) where available, were retained for exploratory comparison.

Each observation represented a sting-related clinical encounter on a given calendar day. Multiple presentations on the same day were counted as a single sting day, whereas events occurring on different days were treated as independent observations, irrespective of patient identity. Individual-level longitudinal tracking was not available; therefore, repeat visits by the same individual across different days could not be distinguished. Accordingly, the unit of analysis throughout the study was the calendar day with ≥1 clinically treated sting event (“sting day”), rather than individual patients or absolute sting counts.

Inclusion criteria comprised all consecutive sting-related encounters requiring medical evaluation during the study period. No exclusions were made based on age or sex. The cohort included patients of White ethnic background only (investigator-observed), reflecting the local hospital catchment population during the study period. Ethnicity was not used as a predictor variable and was not modeled analytically; however, this demographic restriction limits generalizability and is explicitly acknowledged as a source of potential selection bias.

Because the study spanned a 17-year period, changes in clinical documentation systems and recording practices over time cannot be excluded. To reduce heterogeneity, case identification relied on standardized diagnostic coding and core hospital records that were consistently available throughout the observation period. Only variables available in a comparable format across the entire study period were included in the analysis. Information that was not systematically documented, including precise sting location, individual area of residence, urban versus rural exposure context, occupational or recreational activity, detailed exposure circumstances, and species-level identification, was not imputed and is acknowledged as a limitation of the retrospective design.

### 2.2. DWD-Archived Meteorological Data

Step 2 focused on extracting meteorological data from the “Deutscher Wetterdienst” (DWD, German Meteorological Service) [22], a validated source of long-term climate observations [23,24,25]. Data were obtained from one weather station located within the city of Bochum and three additional stations within the surrounding metropolitan area (Table 1).

Daily meteorological values were spatially aggregated using arithmetic means across all available stations for each parameter and day. This approach was chosen to reduce single-station measurement noise and to approximate average environmental conditions across the hospital’s urban catchment area, for which precise sting locations were not available. All sting events were therefore implicitly assumed to occur within the broader metropolitan region covered by the selected stations, an assumption addressed as a limitation.

Missing values were imputed only for parameters showing high inter-station correlation, using same-day averages from neighboring stations. No uncertainty propagation was applied, and imputed values were treated as point estimates.

### 2.3. Correlation Analyses

Step 3 investigated associations between sting occurrence and meteorological conditions following standardized preprocessing of the weather data. The purpose of this step was exploratory feature construction to translate heterogeneous meteorological measurements into a structured indicator framework suitable for subsequent modeling, rather than causal inference.

Five daily meteorological variables with established relevance for insect activity were considered: (i) precipitation, (ii) sunshine duration, (iii) mean cloud cover, (iv) mean wind speed, and (v) mean air temperature. For each variable, indicator scores were computed separately for the current season and for the preceding year (carry-over conditions), yielding a total of ten weather-related indicators x_k,i_. This design aimed to capture both short-term modulation of insect activity and delayed ecological effects on population dynamics.

**Figure 1:**
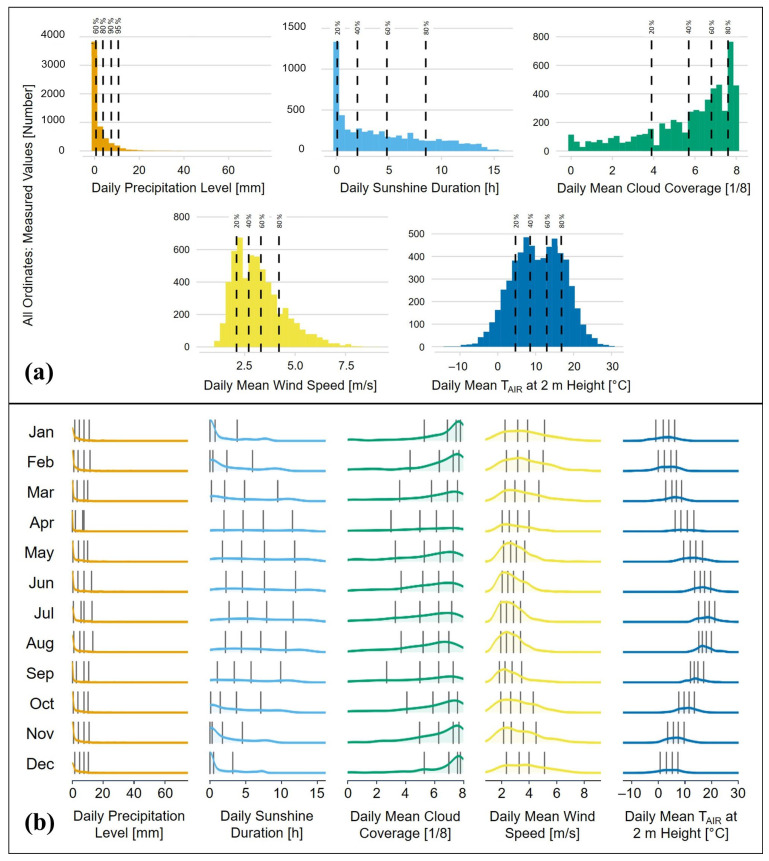
Classification and density distributions of meteorological parameters: (**a**) Quantile-based classification of selected daily weather variables for the calculation of indicator scores defining the “insect year.” Except for precipitation, histograms were stratified into 20% quantile intervals. These empirical distributions were used to differentiate between meteorologically favorable and unfavorable insect activity years. All data were obtained from the *Deutscher Wetterdienst* (DWD) and processed within the machine learning workflow. (**b**) Monthly density distributions of the same parameters. Vertical lines indicate quantile thresholds as defined in (**a**). Monthly aggregation was applied to account for the circannual variability of the depicted meteorological variables.

Daily values were categorized into quantiles derived from the long-term empirical distribution across the full study period: A (0–20%), B (20–40%), C (40–60%), D (60–80%), and E (80–100%) (Figure 1a). For precipitation, skewed distributions required modified thresholds (A: 0–60%, B: 60–80%, C: 80–90%, D: 90–95%, and E: 95–100%). All quantile thresholds were defined a priori and applied uniformly across years.

To account for inter-annual seasonal dynamics (e.g., temperature variation), data were additionally grouped on a monthly basis (Figure 1b). Aggregated indicators were calculated separately for *V. germanica* and *A. mellifera*. Indicator values were computed on a daily basis using weather patterns from the beginning of each year up to the respective reference date, thereby integrating both short-term and carry-over meteorological effects.

Each individual indicator *x_k,i_* was mapped to an ordinal activity score ranging from –2 (weather conditions strongly unfavorable for wasp activity) to +2 (weather conditions strongly favorable) for each day. The aggregated daily indicator *d_A,i_* (exemplary for quantile A: see above) was calculated as the sum of the ten indicator scores according to Formula (1), yielding a theoretical range from −20 (maximally unfavorable) to +20 (maximally favorable conditions).

(1) $$d_{A,i}=\overset{10}{\underset{k=1}{\sum }}x_{k,i}$$

Based on the calculated onset of the sting season for *V. germanica*, the seasonal distribution of favorable and unfavorable conditions was summarized using a weighted and normalized indicator according to Formula (2):

(2) $${\mathrm{Indicator}}_{i}=\frac{-2\cdot d_{A,i}-1\cdot d_{B,i}+0\cdot d_{C,i}+1\cdot d_{D,i}+2\cdot d_{E,i}}{d_{A,i}+d_{B,i}+d_{C,i}+d_{D,i}+d_{E,i}}$$

In this formulation, the number of days assigned to each quantile category was weighted by its corresponding activity score and normalized by the total number of days, yielding a seasonal favorability index ranging from −2 to +2. This normalization enabled comparison of seasonal condition profiles across years.

This rule-based scoring scheme represents an expert-informed feature transformation intended to standardize heterogeneous meteorological inputs; it was not optimized, weighted, or learned from outcome data. Uncertainty propagation for indicator construction was not modeled and is acknowledged as a limitation. Based on this ten-indicator framework, a machine learning model was subsequently developed that incorporated both current-season and preceding-year meteorological information to explore associations with sting occurrence.

### 2.4. Machine Learning for Insect Sting Prediction

Step 4 applied a supervised machine learning approach to explore time-resolved risk of clinically treated Hymenoptera-related sting events based on linked weather and sting-event data. The availability of daily sting counts facilitated initial supervised model development. Initial model development explored daily binary classification of sting versus non-sting days using a multilayer perceptron (MLP), a feedforward neural network. Because daily prediction proved unstable in this rare-event setting, the final modeling framework used weekly aggregation and classified calendar weeks into ordinal risk categories. For benchmarking, we additionally evaluated the clinically relevant binary distinction between low-risk weeks and elevated/high-risk weeks. For training algorithm details, see Supplementary Materials (Figure S1) and reference [26]. Model optimization prioritized accurate identification of elevated- and high-risk periods. Model performance was evaluated primarily using the Recall Score (sensitivity), which quantifies the proportion of actual elevated-risk periods correctly identified, as defined by Formula (3):

(3) $$\mathrm{Recall}\;\mathrm{Score}=\frac{\mathrm{tp}}{\mathrm{tp}+\mathrm{fn}}$$

where *tp* denotes true positives, defined as correctly identified elevated- or high-risk periods, and *fn* denotes false negatives, defined as elevated- or high-risk periods not identified by the model. The Recall Score ranges from 0, indicating that no elevated- or high-risk periods were correctly identified, to 1, indicating that all elevated- or high-risk periods were correctly identified.

It should be noted that only a subset of sting events (*n* = 138) could be assigned to a confirmed insect species over the 17-year observation period. Consequently, the present machine learning approach was not designed to support species-specific prediction models. Instead, sting events were analyzed in an aggregated manner to explore general, time-resolved risk patterns across Hymenoptera-related stings.

Given the limited number of clinically significant sting events, the MLP architecture was intentionally kept shallow, and hyperparameter optimization was restricted to a predefined parameter space. Feature engineering was performed before model fitting using predefined meteorological and temporal variables, including calendar-derived variables, daily and weekly aggregated weather parameters, current-season and preceding-year indicator scores, and cumulative seasonal sunshine duration. Indicator construction was based on expert-informed ecological assumptions and empirical weather distributions. However, the final season-onset and risk-threshold definitions were treated as exploratory because they were refined within the available dataset.

Model development followed a train–test separation strategy. Hyperparameter tuning was performed only within the training data using five-fold cross-validation. The evaluated MLP hyperparameters included activation function, solver, learning-rate schedule, and regularization strength (α). Preprocessing steps, including imputation and scaling, were fitted within the training data and applied unchanged to validation or test data to reduce information leakage. The independent test data were not used for hyperparameter selection. Because indicator construction and threshold refinement were partly outcome-informed, all performance estimates were interpreted as exploratory rather than confirmatory.

### 2.5. Statistical Analysis and Software

Statistical analyses were conducted to describe the distribution of sting events, characterize class imbalance, and evaluate model performance. The primary unit of analysis was the calendar day or calendar week, depending on the prediction framework. Continuous variables are reported as means with standard deviations or medians with interquartile ranges, as appropriate. Categorical variables are summarized as counts and proportions.

Model performance was assessed using confusion-matrix-derived metrics, including recall (sensitivity), specificity, precision, negative predictive value, F1-score, and overall accuracy. Given the pronounced imbalance between sting and non-sting periods, model optimization prioritized recall for elevated- and high-risk periods in order to minimize false-negative predictions. Additional performance metrics were reported to quantify false-positive burden and to contextualize clinical interpretability under class imbalance.

To contextualize the added value of the MLP, we performed a post hoc baseline benchmarking analysis using the same weekly prediction framework and the existing held-out test split. The primary binary endpoint was an elevated/high-risk week versus a low-risk week. Comparator approaches included a majority-class classifier, a fixed calendar-season rule, historical calendar-week prevalence estimated from the training set, calendar-only logistic regression, weather-enhanced logistic regression, indicator-enhanced logistic regression, and a shallow decision tree. Thresholds for probabilistic baseline models were selected exclusively within the training data to reduce test-set leakage. Model performance was evaluated on the held-out test set using recall, specificity, precision, negative predictive value, F1-score, and overall accuracy. A complementary three-class evaluation was performed for low-, elevated-, and high-risk weeks.

Model optimization was performed within the weekly prediction framework using five-fold cross-validation. No synthetic oversampling or resampling techniques were applied, as these may artificially inflate rare-event patterns in small datasets. Performance estimates are reported descriptively. No formal hypothesis testing or statistical inference regarding predictive superiority was performed, as the analysis was designed as a feasibility and proof-of-concept study rather than a confirmatory modeling exercise. All analyses were performed using Python software version 3.12.3 (Python Software Foundation, Beaverton, OR, USA) and R software version 4.4.3 (R Foundation for Statistical Computing, Vienna, Austria), with workflow orchestration via Jupyter Notebook version 7.3.1 (Project Jupyter). Data processing relied primarily on pandas version 2.2.3, visualization on ggplot2 version 3.5.2, and machine learning on scikit-learn version 1.6.1 in Python [27].

Source code was modularized into annotated code blocks using markdown cells, allowing transparent and stepwise evaluation of data processing and modeling stages. All software tools used were open-source. Additional details are provided in the Supplementary Material File S1. This study was reported in accordance with the STROBE guidelines for observational studies.

## 3. Results

### 3.1. Clinically Significant Insect Sting Events

**Figure 2:**
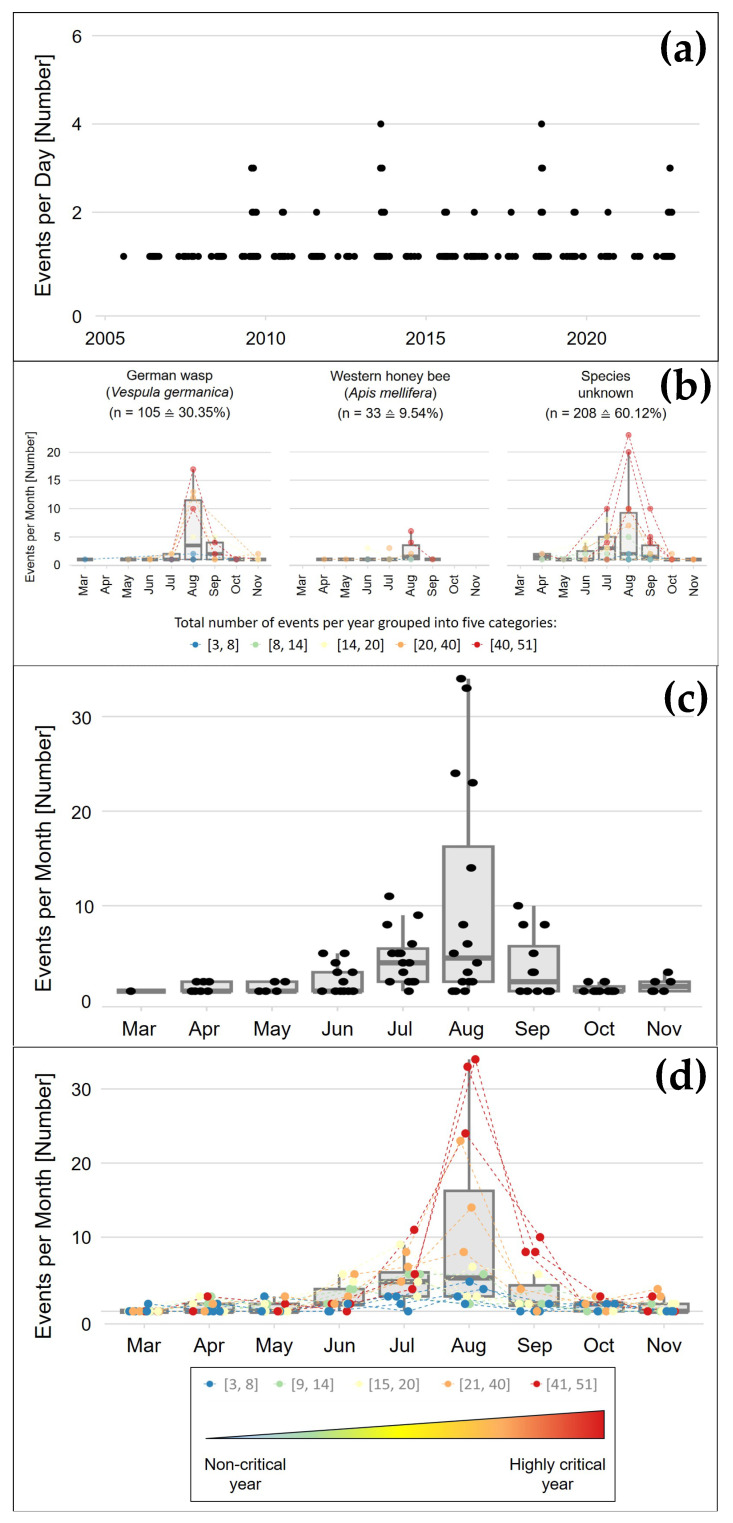
Temporal distribution of clinically recorded insect sting events: (**a**) Time-series representation of insect sting events recorded during the observation period. The 346 events shown cover data collected between 31 July 2005 and 31 July 2022. Only a few days exhibited more than one sting requiring medical treatment. Regular gaps along the one-event-per-day line reflect the pronounced seasonality, with no sting events recorded during winter months. (**b**) Monthly distribution of sting events stratified by insect species, with color coding indicating categorized annual sting frequencies (no events were recorded in January, February, or December). (**c**). Event counts per month for all fully documented years; individual data points represent monthly values from different calendar years (no events were recorded in January, February, or December). (**d**) Aggregated monthly representation of the entire observation period. Years were classified into five categories ranging from non-critical to highly critical, based on total annual sting frequencies (color code shown at the bottom, with minimum and maximum values indicated in square brackets). Dashed lines connect data points originating from the same calendar year.

A total of 346 clinically significant insect sting events requiring medical attention were recorded at the University Hospital over the 17-year observation period (Figure 2a). Species attribution was available for 138 events (39.9%), including 105 wasp stings and 33 bee stings, while 208 events (60.1%) lacked explicit species documentation and were classified as species unknown (Figure 2b). These unidentified cases were retained in the analysis as clinically relevant sting events but were not assumed to represent a specific insect species. Consequently, all subsequent analyses focused on aggregate sting occurrence rather than species-specific inference. Sting events occurred between March and November, with a pronounced seasonal peak during June to September, consistent across most years (Figure 2c). Considerable inter-annual variability was observed, with some years exhibiting >30 events during peak summer months, whereas others showed minimal activity. Based on observed monthly sting event counts, calendar years were descriptively categorized into five incidence groups ranging from non-critical to highly critical. The grouping was defined a priori using absolute monthly event count ranges to facilitate visual comparison of seasonal patterns rather than formal statistical classification. Specifically, years were assigned to the following categories based on their maximum monthly sting counts: 3–8 events (non-critical), 9–14 events (low), 15–20 events (moderate), 21–40 events (critical), and 41–51 events (highly critical) (Figure 2d). These categories were used solely for descriptive visualization and were not employed as outcome labels or predictors in subsequent modeling. During the months of March to June and October to November, the number of events showed only negligible variation between highly critical (2013 and 2018), critical (2009 and 2022), and non-critical years. The most pronounced differences in observed event counts between highly critical and non-critical years occurred in August and September (Figure 2d).

### 3.2. Meteorological Data Availability and Station Agreement

**Figure 3:**
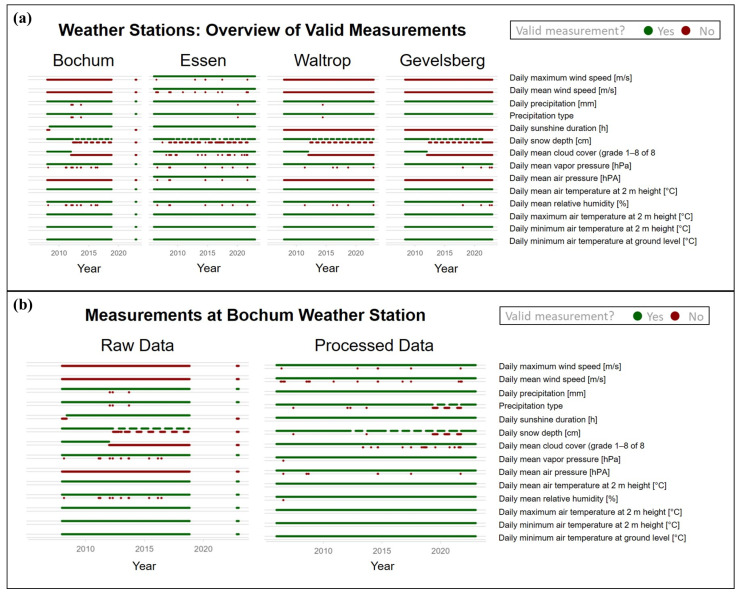
Availability and completeness of meteorological measurements: (**a**) Time-resolved overview of available measurement series for the four meteorological stations included in the analysis. Periods with missing entries indicate intervals during which no valid data were available. Data status is shown after initial quality control and preprocessing, as described in Section 2. Across stations, most measurement series are largely complete over the observation period. Valid measurements are indicated in green and invalid or missing measurements in red. (**b**) Measurement availability at the Bochum weather station. Shown are data before (raw data) and after preprocessing, illustrating the generation of a near-complete time series for the full observation period. Valid measurements are indicated in green and invalid or missing measurements in red.

Several commercial providers offer weather data accessible via an Application Programming Interface (API) [28]; however, a pilot evaluation revealed frequent temporal gaps and inconsistent parameter availability. Therefore, meteorological data from the DWD were selected, as these data undergo standardized quality control procedures and represent an established reference for climatological analyses in Germany [25,29]. Daily meteorological measurements were obtained from one weather station located within the city of Bochum and three additional stations in the surrounding metropolitan area (Supplementary Materials Figure S2). Across all stations, 14 meteorological parameters were available: 1. wind speed (daily maximum value [m/s]); 2. wind speed (daily mean value [m/s]); 3. precipitation (daily amount [mm]); 4. precipitation type (various); 5. sunshine (daily duration [h]); 6. snow (daily depth [cm]); 7. mean cloud cover (daily grades 1–8 of 8); 8. mean vapor pressure (daily value [hPa]); 9. mean air pressure (daily value [hPa]); 10. mean air temperature at 2 m height (daily value [°C]); 11. maximum air temperature at 2 m height (daily value [°C]); 12. minimum air temperature at 2 m height (daily value [°C]); 13. minimum air temperature at ground level (daily value [°C]); and 14. mean relative humidity (daily value [%]) (Figure 3a,b). As shown in Figure 3b, the Bochum station exhibited two major time gaps, indicating periods of non-operation, i.e., prior to 1 January 2008 and from 1 November 2018 to 31 October 2022. Moreover, several measurements are marked as invalid (red labels), so it was sensible to additionally include the three stations in Bochum’s greater metropolitan area (Table 1; Figure 3a; Supplementary Materials Figure S2).

To systematically examine the 14 parameter measurement series at the weather stations and to improve clarity, the parameters were grouped into five clusters: (i) temperature (cluster A); (ii) precipitation/snowfall (cluster B); (iii) wind conditions (cluster C); (iv) sunshine/cloud cover (cluster D); and (v) humidity/air pressure (cluster E). Each of these clusters was evaluated to assess the data plausibility and the degree of correlation among measurements from different stations. As expected for stations located within the same metropolitan region and exposed to shared seasonal forcing, high Pearson correlations were observed within parameter clusters across stations. For cluster A (Supplementary Materials Figure S2), the Pearson’s correlation coefficient (*R*) ranged from 0.95 to 1.00 (*p* < 2.2 × 10^–16^), and for cluster B (Supplementary Materials Figure S3), *R* ranged from 0.66 to 0.95 (*p* < 2.2 × 10^–16^). Wind measurements (cluster C) were only taken at the station in Essen-Bredeney, showing a plausible yearly trend, with slightly weaker winds in summer compared to fall and winter (Supplementary Materials Figure S4). For cluster D (Supplementary Materials Figure S5), *R* ranged from 0.83 to 0.98 (*p* < 2.2 × 10^–16^), and for cluster E (Supplementary Materials Figure S6), it ranged from 0.93 to 0.95 (*p* < 2.2 × 10^–16^). These correlations primarily indicate redundancy and spatial coherence, rather than independent confirmation of measurement accuracy.

Based on this station agreement, missing values at the Bochum station were supplemented using daily averages from neighboring stations for parameters demonstrating strong inter-station correlation. Temperature values were averaged across all four stations, wind data were obtained from the Essen-Bredeney station, and precipitation-, sunshine-, and pressure-related variables were imputed using metropolitan averages when local data were unavailable. Precipitation type was inferred from combined precipitation and snowfall measurements.

This approach resulted in a near-complete daily meteorological dataset spanning the full 17-year observation period (see processed data in Figure 3b). However, imputed values were treated as point estimates, and the variance introduced by imputation was not explicitly modeled. Consequently, reconstructed exposure series should be interpreted as approximations of regional conditions rather than exact local measurements. The potential impact of residual exposure misclassification on downstream analyses is therefore acknowledged as a limitation.

### 3.3. Exploratory Estimation of Wasp Season Onset Using Weather-Based Indicators

To explore whether seasonal timing of insect activity is associated with sting occurrence, an exploratory, parameter-based estimation of wasp season onset was performed based on established ecological knowledge of wasp life cycles. In particular, the emergence of overwintering queens in spring has been described as a key indicator for the start of the wasp season [4]. Building on this ecological framework, weather-derived indicators were constructed to characterize favorable and unfavorable conditions for insect activity, using historical meteorological data. These indicators were not intended to provide causal inference, but rather to capture seasonal structure and timing that could plausibly modulate sting occurrence. The resulting season-onset estimates were subsequently used as contextual features within the broader modeling framework.

**Figure 4:**
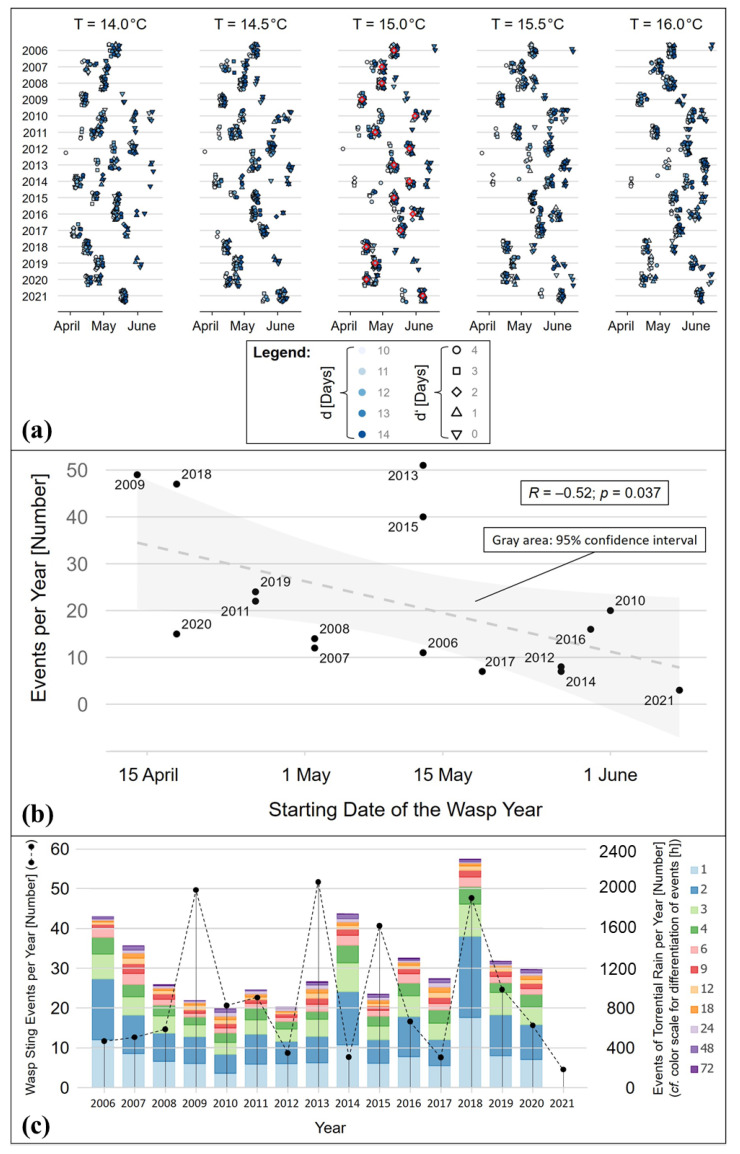
Wasp season onset, wasp year definition, and annual sting incidence: (**a**) Calculated onset of the wasp season, defining the beginning of the “wasp year” based on the emergence/awakening of queen wasps in spring. Shown are calculated start dates for all tested parameter combinations of *d*, *d*′, and *T*. Estimated onset dates predominantly fall within April to May, with year-specific clustering. Red symbols highlight the selected parameter set (*d* = 12, *d*′ = 2, *T* = 15 °C), representing the median values of the tested parameter ranges. (**b**) Association between the calculated onset date of the wasp year and the total number of sting events per year. Annual sting counts are plotted against the corresponding starting dates of the wasp season. The dashed line represents the linear regression fit, and the gray area indicates the 95% confidence interval. Earlier season onset is associated with higher annual sting numbers. The overall correlation, quantified using Pearson’s correlation coefficient (R = −0.52; *p* = 0.037), indicates a statistically significant inverse relationship despite the presence of outlier years (e.g., 2013 and 2015). (**c**) Evaluation of the relationship between annual wasp sting frequencies and torrential rain events. Shown are annual numbers of wasp sting events (2006–2020; left ordinate) and annual numbers of torrential rain events (2006–2021; right ordinate). The black dashed line with dots represents annual wasp sting events. According to the Deutscher Wetterdienst (DWD), torrential rain is defined as precipitation exceeding 40 L/m^2^ within 1 h or 60 L/m^2^ within 6 h (or multiples thereof). The eleven duration categories of torrential rain events (1–72 h) are color-coded. In line with DWD classifications, precipitation events were predominantly convective. Despite interannual variability and several extreme years, an overall increase in the annual number of torrential rain events was observed.

Using a parameter-based approach, this study defines the onset of the wasp season by identifying temperature thresholds over specific durations—e.g., ~15 °C sustained for at least 10–14 days. To increase precision, the definition of the wasp season onset was refined using exact temperature and duration parameters. The final definition was as follows: The wasp season begins when, for the first time in a given year, over a period of d + d′ days, the daily maximum temperature reaches at least T degrees Celsius for d of those days. For example, if d = 12, d′ = 2, and T = 15 °C, the requirement is met when a temperature of ≥15 °C is reached on ≥12 days within a 14-day period. This parameter-dependent definition allows testing the robustness of results across different values of the three parameters: d ∈ (10, 11, 12, 13, 14), d′ ∈ (0, 1, 2, 3, 4), and T ∈ (14, 14, 5, 15, 15, 5, 16) °C. This resulted in 125 parameter combinations and 2000 total onset estimates across the 16-year observation period. The variant highlighted in Figure 4a (i.e., d = 12, d′ = 2, T = 15 °C) produced onset estimates that qualitatively aligned with years exhibiting higher sting frequencies. This parameter set was therefore selected post hoc for the AI model to define the onset of the wasp season. This selection therefore represents an outcome-informed, exploratory procedure rather than an independent validation.

When annual sting counts were plotted against the estimated season onset dates (Figure 4b), earlier onset was associated with higher total sting numbers (Pearson R = −0.52; *p* = 0.037). However, this association was derived after parameter tuning and was not validated on independent data, and several years deviated from the overall trend. Accordingly, this finding should be interpreted as hypothesis-generating, suggesting that seasonal timing may influence the temporal distribution of sting events without serving as a stand-alone predictor of annual sting incidence.

The season-onset estimates were therefore used only as contextual inputs within the subsequent machine learning analysis, rather than as definitive predictors of sting risk.

### 3.4. Machine Learning-Based Risk Stratification

Step 4: An AI-based approach was applied to predict insect stings on a daily basis. The dataset was prepared accordingly (Supplementary Materials Figure S1), and the model was optimized using a grid search method. This involved systematically varying all parameters of the machine learning model in order to identify the most suitable configuration for prediction. Daily prediction proved insufficiently precise due to the limited number of events and inherent stochasticity; therefore, weekly risk assessment was implemented. Attributes were aggregated into average weekly values, and calendar weeks replaced month identifiers. Labels were grouped into three ordinal risk levels: low (no stings), elevated (one sting), and high (≥2 stings). These categories were defined a priori to reflect increasing clinical relevance, rather than being optimized post hoc. Cross-validation prioritized elevated and high-risk weeks using recall as the primary metric to select models that effectively identified critical weeks. The initial cross-validation iteration yielded a recall of 0.347 for the model variant (identity, L-BFGS, constant), where L-BFGS refers to the limited-memory Broyden–Fletcher–Goldfarb–Shanno algorithm. Subsequent variation of the regularization parameter α (0.00001–0.0005) improved recall to 0.431, with an optimal α of 0.0005.

Additional performance metrics were derived from cross-validated predictions to contextualize model reliability under pronounced class imbalance. Weekly model performance showed a specificity of 0.86, a negative predictive value of 0.91, a precision of 0.31, an F1-score of 0.33, and an overall accuracy of 0.82. These values indicate that the model primarily identified low-risk weeks, whereas precision for elevated- and high-risk weeks remained limited.

To evaluate whether the MLP provided added value beyond simpler approaches, we performed a post hoc benchmark analysis using the held-out test split and the same weekly prediction framework. For the binary distinction between low-risk and elevated/high-risk weeks, the MLP did not consistently outperform simpler baseline models (Table 2). On the held-out test split, the MLP achieved a recall of 0.568, a specificity of 0.847, a precision of 0.556, a negative predictive value of 0.854, an F1-score of 0.562, and an accuracy of 0.777. Several simpler comparator models achieved higher recall and/or F1-scores than the MLP. In particular, historical calendar-week prevalence, weather-enhanced logistic regression, indicator-enhanced logistic regression, and the shallow decision tree showed higher F1-scores, while the shallow decision tree and indicator-enhanced logistic regression achieved the highest recall values. These findings indicate that the MLP did not provide consistent added value over simpler baseline approaches for the binary warning task.

In the complementary three-class ordinal evaluation, the MLP achieved the highest macro F1-score among the tested models (Supplementary Material Table S1), suggesting potential exploratory value for differentiating low-, elevated-, and high-risk weeks (ordinal risk differentiation), rather than for binary warning. Taken together, these findings suggest that weather-informed modeling can identify a subset of elevated short-term sting-risk periods. However, benchmarking against simpler baselines indicates that the MLP should be interpreted as an exploratory proof-of-concept rather than as a clearly superior predictive model.

## 4. Discussion

The present work advances the field by combining long-term, clinically validated sting records with high-resolution weather data and machine learning to quantify short-term exposure risk in a rare-event context. This novel integration allows for identification of intra-seasonal clustering patterns, providing insights that extend beyond descriptive epidemiology and may inform future risk-awareness tools.

Hymenoptera venom represents one of the most frequent triggers of anaphylaxis in adults and remains a clinically relevant public health concern [30,31]. In Germany alone, several million individuals are considered at risk for systemic sting reactions [4], underlining the importance of preventive strategies that aim to reduce exposure rather than treating allergic reactions after they occur.

The present study explored whether routinely available meteorological data can be linked to hospital-treated insect sting events to enable time-resolved risk stratification. Using a long-term, single-center dataset combined with regional weather observations, we evaluated the feasibility of a weather-informed, machine learning-based approach to identify periods of increased sting occurrence. Importantly, the outcome modeled in this study was clinically treated sting events, which serve as a pragmatic proxy for exposure requiring medical attention, rather than a direct measure of allergy incidence or anaphylaxis risk.

Our findings indicate that meteorological parameters are associated with temporal clustering and intra-seasonal variation of sting events, rather than with absolute annual sting counts. Weekly aggregation enhanced recall compared with daily prediction, reflecting the stochastic nature and rarity of individual sting events. The cross-validated recall of 0.431 suggests that the model was able to identify a subset of elevated-risk weeks; however, this performance must be interpreted cautiously in light of the low prevalence of events and the corresponding trade-off between sensitivity and false-positive burden.

Consistent with the reported performance metrics, the model’s discriminative capacity was primarily driven by reliable identification of low-risk periods rather than precise prediction of elevated-risk weeks. This pattern reflects pronounced class imbalance and deliberate prioritization of sensitivity in a rare-event setting, resulting in an expected increase in false-positive classifications and modest precision. Accordingly, the present analysis demonstrates technical feasibility rather than predictive effectiveness and supports an exploratory, exclusion-oriented approach to risk stratification rather than a validated or confirmatory forecasting tool.

The baseline benchmarking analysis further qualifies these findings. Although the MLP captured some ordinal structure in weekly risk categories, it did not consistently outperform simpler seasonal or statistical approaches for the binary warning task. This finding argues against interpreting the MLP as a superior stand-alone prediction model. Rather, the present analysis should be viewed as a feasibility study demonstrating that routinely collected clinical sting data can be linked with meteorological information to support exploratory, time-resolved risk stratification.

Notably, despite the study period coinciding with a phase of rising ambient temperatures [32], no consistent association was observed between annual sting counts and long-term temperature trends or extreme precipitation events [33]. This finding underscores that meteorological variables in this dataset predominantly influenced intra-seasonal timing and clustering of sting events rather than acting as linear drivers of annual incidence. However, given the single-region design and limited event numbers, these observations should not be interpreted as evidence against broader climate-related effects.

Beyond the immediate findings, the present work provides a conceptual framework that may be adaptable to other medically relevant insect species if supported by appropriate ecological and exposure data. In this context, species-specific information from established insect-monitoring networks and radar-based surveillance systems could complement clinical datasets in future studies, particularly for invasive Hymenoptera species associated with increased medical risk [8,34].

This study provides several relevant insights. First, it supports the concept that meteorological conditions primarily modulate the timing and short-term distribution of sting events within a season. Second, it demonstrates that weekly aggregation is more suitable than daily prediction in data-scarce settings. Third, it highlights key methodological challenges—event rarity, exposure misclassification, and behavioral confounding—that must be addressed before any practical deployment can be considered.

More broadly, recent perspectives have highlighted the growing role of AI in allergology, particularly for diagnostic and therapeutic applications [35]. The present study illustrates a potential preventive extension of such approaches. For practical translation, however, the proposed framework should be viewed as a risk-awareness layer rather than as a stand-alone warning system. In a future implementation, such a tool could support time-resolved communication of periods with elevated environmental sting risk, for example, in allergy clinics, emergency departments, public health messaging, or for individuals with a known Hymenoptera venom allergy.

Clinical or public health implementation would require prospective external validation in larger, multi-institutional datasets covering different geographic regions, climatic conditions, healthcare settings, and insect-exposure patterns, including regions with higher Hymenoptera exposure and sting incidence, such as Mediterranean settings [36,37]. Such datasets should ideally combine standardized clinical sting-event documentation with harmonized meteorological data and additional behavioral and ecological covariates, including outdoor activity patterns, land use, green-space exposure, occupational or recreational exposure, and, where available, insect activity or surveillance data. Before implementation, acceptable performance thresholds would need to be predefined according to the intended use case, with particular emphasis on high sensitivity for rare sting-event periods, adequate calibration, and an acceptable false-alert burden. Therefore, the present findings should be interpreted as a methodological basis for future risk-awareness tools, including potential mHealth applications, rather than as evidence supporting immediate clinical or public health deployment [38,39,40,41,42,43,44].

### 4.1. Limitations

This study has several important limitations that should be considered when interpreting the findings. First, the outcome measure was based on hospital-treated insect sting events, which are influenced not only by insect activity but also by healthcare-seeking behavior, access to medical care, and potential changes in admission, diagnostic, or documentation practices over time. As individual-level linkage was unavailable, repeated presentations by the same individual could not be identified, and relevant exposure contexts such as occupational, recreational, or environmental activities were not captured. Consequently, the modeled outcome reflects a composite of environmental exposure and healthcare utilization rather than true sting incidence in the general population, and temporal clustering or repeat presentations cannot be excluded.

Second, the study was conducted at a single center within a single urban region and relied on a relatively small number of clinically treated sting events collected retrospectively, limiting statistical power and generalizability. Species attribution was incomplete, precluding species-specific modeling and restricting inference to aggregated Hymenoptera-related sting events. Meteorological exposure was reconstructed using data from multiple nearby weather stations, and missing values were conservatively imputed only for parameters with high inter-station correlation. Imputed values were treated as point estimates without formal uncertainty propagation, introducing potential exposure misclassification.

Third, several components of the modeling pipeline—including indicator construction, season-onset definitions, and risk thresholds—were refined using the same dataset later employed for model evaluation. Although cross-validation and partial temporal separation were applied to mitigate overfitting, this outcome-informed optimization may have inflated apparent performance. Although a post hoc benchmark analysis against simpler baseline models was performed, this comparison was conducted within the same single-center retrospective dataset and does not replace external validation. In addition, no external validation dataset was available. Therefore, the scope of generalizability is currently limited to comparable urban Central European settings, and extrapolation to rural regions, high-exposure areas, different healthcare systems, or species-specific sting risks requires prospective validation. Future validation should therefore include diverse, multi-institutional datasets from regions with different climatic conditions, insect populations, healthcare structures, and exposure patterns to assess the generalizability and transportability of the proposed framework. Accordingly, the current findings should be regarded as exploratory rather than confirmatory.

Finally, from a clinical perspective, the achieved recall values underscore a fundamental limitation for practical use. For a potentially life-threatening condition such as Hymenoptera venom anaphylaxis, missing a substantial proportion of high-risk periods would be unacceptable if the model were used as a stand-alone decision tool. Although weekly aggregation improved sensitivity compared with daily prediction, it did not overcome this constraint. The proposed model is therefore not intended to replace established preventive measures or clinical decision-making but rather to function as a complementary risk-awareness tool to inform precautionary behavior during periods of elevated environmental risk.

### 4.2. Conclusions

In summary, this study demonstrates the feasibility of linking long-term clinical insect-sting data with meteorological information for exploratory, time-resolved risk stratification. By integrating environmental and clinical data, the study provides a methodological framework for examining short-term sting-risk patterns in a rare-event context. The findings indicate that weather-related factors primarily influence the intra-seasonal timing and clustering of clinically treated sting events rather than absolute annual incidence.

Importantly, the results do not establish predictive effectiveness and are not intended to support immediate clinical, public health, or policy applications. Post hoc benchmarking showed that the MLP did not consistently outperform simpler baselines for binary warning of elevated-risk weeks. The model’s potential value appears to lie primarily in ordinal risk differentiation and hypothesis generation rather than in stand-alone binary warning.

Future studies should focus on external validation in larger, multi-center cohorts, prospective calibration, comparison across regions with higher sting incidence, and integration of behavioral, ecological, and exposure-related covariates. Only through such rigorous evaluation can the clinical relevance and practical utility of time-resolved sting risk-awareness tools be reliably determined before operational, clinical, or public health use can be considered.

## Figures and Tables

**Table 1:** Meteorological stations of the “Deutscher Wetterdienst” (DWD; German Meteorological Service) used in this study.

Location	ID	Height Above Mean Sea Level [m]	Operating Since
City of Bochum	555	110	1 January 1940
Bochum metropolitan region:			
– Essen-Bredeney	1303	150	1 January 1935
– Waltrop-Abdinghof	13696	60	1 December 2007
– Gevelsberg-Oberbroeking	13700	203	1 May 2008

Note: Weather data were obtained from the primary station in Bochum and three surrounding metropolitan stations to ensure data completeness across the observation period. Station altitude and start of operation are reported to document data availability and comparability.

**Table 2:** Post hoc benchmark comparison for the binary distinction between low-risk and elevated/high-risk weeks on the held-out test split.

Model	Recall	Specificity	Precision	NPV	F1-Score	Accuracy
MLP	0.568	0.847	0.556	0.854	0.562	0.777
Majority-class classifier/always low risk	0.000	1.000	NA	0.749	0.000	0.749
Fixed calendar-season rule	0.773	0.748	0.507	0.907	0.613	0.754
Historical calendar-week prevalence	0.750	0.794	0.550	0.904	0.635	0.783
Calendar-only logistic regression	0.614	0.802	0.509	0.861	0.557	0.754
Weather-enhanced logistic regression	0.659	0.840	0.580	0.880	0.617	0.794
Indicator-enhanced logistic regression	0.795	0.740	0.507	0.915	0.619	0.754
Shallow decision tree	0.841	0.695	0.481	0.929	0.612	0.731

Note: The binary endpoint was defined as elevated/high-risk week versus low-risk week. All models were evaluated using the same held-out test split. MLP, multilayer perceptron; NPV, negative predictive value; NA, not applicable because the majority-class classifier generated no positive predictions, resulting in an undefined precision estimate.

## Data Availability

Data supporting the findings of this study can be obtained from the corresponding author upon reasonable request and in accordance with institutional and ethical regulations.

## Supplementary Materials

The following supporting information can be downloaded at: https://www.mdpi.com/article/10.3390/ijerph23070881/s1.

## Abbreviations

AI	artificial intelligence
API	Application Programming Interface
CI	confidence interval
DWD	Deutscher Wetterdienst (German Meteorological Service)
ED	emergency department
ICD	International Statistical Classification of Diseases and Related Health Problems
IgE	immunoglobulin E
L-BFGS	limited-memory Broyden–Fletcher–Goldfarb–Shanno algorithm
MLP	multilayer perceptron
mHealth	mobile health
R	Pearson’s correlation coefficient
