# Supplementary material for: Weather-Enhanced Machine Learning for Time-Resolved Risk Stratification of Clinically Managed Hymenoptera-Related Sting Events in an Urban German Region
Source: Int J Environ Res Public Health. 2026 Jul 8;23(7):881. doi: 10.3390/ijerph23070881 (PMC13410303; doi:10.3390/ijerph23070881)
Supplement: Supplementary file 1 [file ijerph-23-00881-s001.zip › ijerph-4364503-supplementary.pdf]

Supplementary information

S1 Fig.

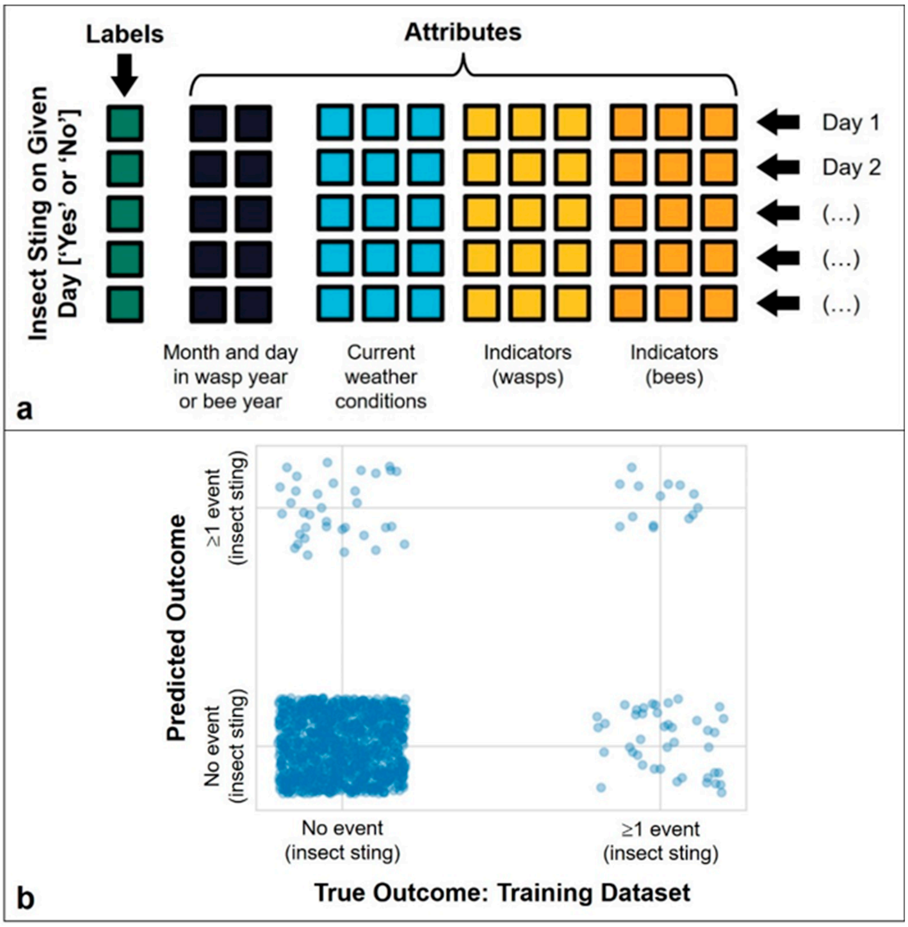

Fig S1. Daily risk assessment of insect stings.

(a) Schematic overview of the dataset structure used for day-specific sting risk prediction. Each row represents a single calendar day within the observation period. The outcome label indicates whether a clinically relevant insect sting occurred on that day and is encoded as a binary variable ("sting" vs. "no sting"). Model prediction was based on a set of attributes comprising: (i) the calendar month and day within the wasp year (defined relative to the calculated seasonal onset) or within the bee year (defined from the beginning of the year for days with a maximum temperature ≥10 °C); (ii) daily meteorological variables processed as described in Step 2; and (iii) derived indicator variables for wasps and bees as defined in Step 3. Cumulative sunshine duration for both the wasp and bee years was included as an additional feature. For machine learning, the dataset was split into training and test sets using a standard 80:20 ratio. Model training and hyperparameter optimization were performed exclusively on the training dataset to prevent data snooping bias. Prior to model fitting, the data were transformed using a preprocessing pipeline that was trained on the training data and applied identically to the test data. The pipeline included:

1. Imputation of missing values, which were rare and replaced by median values
2. Feature scaling of numerical variables to a range between 0 and 1

3. Processing of categorical variables, with the month variable treated numerically (range 1–12).
   As the outcome variable described the presence or absence of insect stings, the task was formulated as a binary classification problem. A multilayer perceptron classifier (MLPClassifier, scikit-learn) was used, and model hyperparameters were systematically optimized using grid search. The evaluated parameter space included:
   - Activation functions ∈ (identity, logistic, tanh, relu)
   - Solvers ∈ (L-BFGS, sgd, adam)
   - Learning rate schedules ∈ (constant, invscaling, adaptive)

(b) Performance evaluation of the daily prediction model. A total of 36 model variants were assessed using five-fold cross-validation. The best-performing configuration was (logistic, L-BFGS, constant), achieving a Recall Score of 0.294. Subsequent optimization of the regularization parameter α by grid search (five-fold cross-validation) identified $\alpha = 0.0001$, but did not further improve recall. The optimized model was then evaluated on the independent test dataset. Although the model correctly predicted 14 days with insect stings, performance was limited by both false-negative and false-positive predictions. Given the rarity of clinically relevant insect stings in the dataset, day-specific prediction proved unreliable, motivating the subsequent aggregation of data to a weekly prediction framework to improve model robustness.

File S1. Further detail on METHODS

Further detail on: **METHODS: *2.5 Statistical analysis and Software***

Machine Learning Tools: Shown in alphabetical order are the employed free-of-charge AI machine learning tools of the MLP neural network model including their sources of supply.

- *ggplot2:* System for creating graphics based on the grammar of graphics in statistics and computing (The R Foundation, an official part of the Free Software Foundation; Boston, MD, USA): https://ggplot2.tidyverse.org
- *Jupyter:* Software, standards, and services for interactive computing across multiple programming languages (Project Jupyter; Berkeley, CA, USA): https://jupyter.org
- *NumPy:* A program library for the *Python* programming language that enables simple handling of vectors, matrices or, generally, large multidimensional arrays. In addition, *NumPy* offers efficiently implemented functions for numerical calculations. (See also under *Scikit-learn*.)
- *Pandas:* Data analysis and manipulation tool built on top of *Python* (Python Software Foundation; Beaverton, OR 97008, USA): https://pandas.pydata.org/
- *Python:* High-level, general-purpose programming language (Python Software Foundation): https://www.python.org/
- *R:* Programming language for statistical computing and data visualization (The R Foundation): https://www.r-project.org/
- *scikit-learn (short for SciPy Toolkit):* Machine learning library for *Python* based on the numerical and scientific *Python* libraries *NumPy* and *SciPy* that offers various classification, regression and clustering algorithms including support vector machines, random forest, gradient boosting (like XGBoost), k-means and DBSCAN (Python Software Foundation): https://scikit-learn.org
- *SciPy:* A *Python*-based open-source software environment for scientific computing, visualization and related activities. Core part of the *SciPy* environment is a *Python* library of numerical and scientific algorithms and mathematical tools. (See also under *Scikit-learn*.)

S2 Fig.

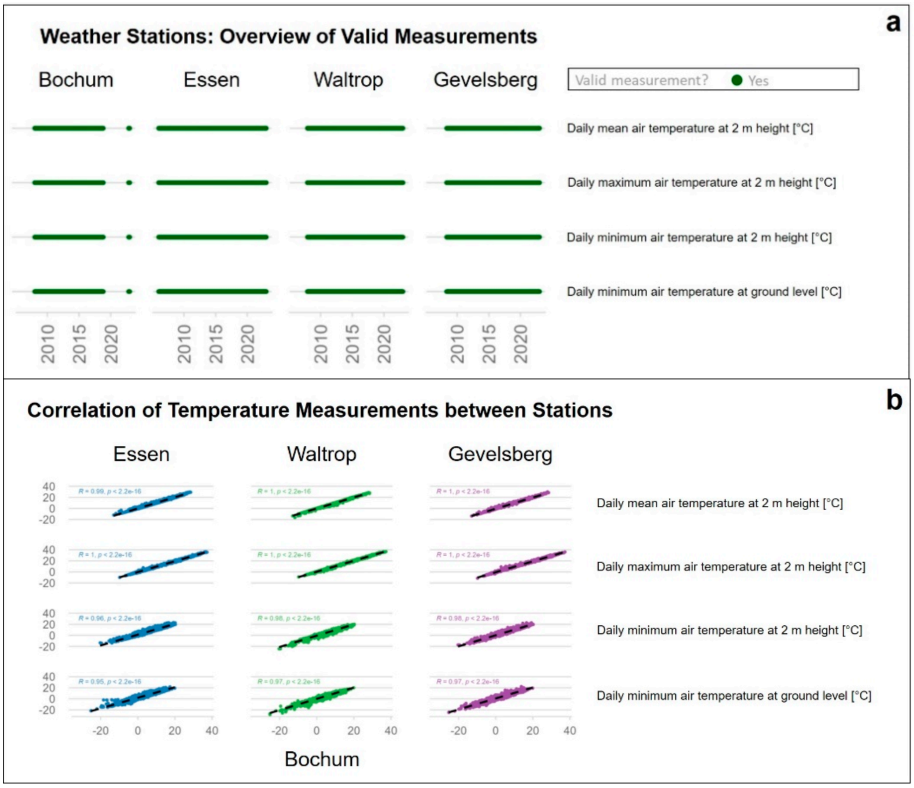

S2 Fig. Cluster A: Temperature measurements.

As described in the Methods section, three additional meteorological stations in the vicinity of Bochum—Essen-Bredeney (Essen), Waltrop-Abdinghof (Waltrop), and Gevelsberg-Oberbröking (Gevelsberg)—were included to supplement the primary station. These stations provide multiple temperature-related measurement series and were therefore well suited to ensure data completeness and cross-station comparability.

(a) Temporal availability of temperature measurements at each station. Periods with missing entries indicate intervals during which no valid data were available.

(b) Correlation analysis of daily temperature measurements between Bochum and each neighboring station, quantified using Pearson’s R, demonstrated strong inter-station agreement across all temperature variables. Each data point represents a single daily measurement.

S3 Fig.

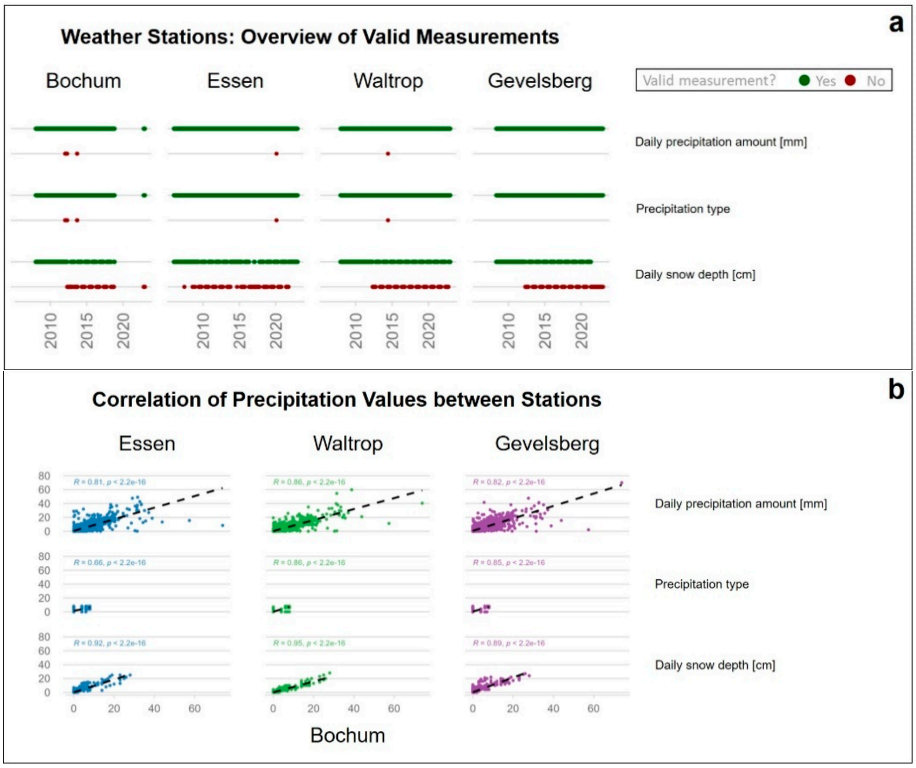

S3 Fig. Cluster B: Precipitation and snowfall measurements.
Measurement series included (i) daily precipitation amount (mm); (ii) daily snow depth (cm); and (iii) precipitation type encoded as a numeric variable (‘0’ no precipitation; ‘4’ precipitation present, form unknown; ‘6’ rain only; ‘7’ snow only; ‘8’ rain and snow and/or sleet; ‘9’ measurement error).

(a) Temporal availability of precipitation-related measurements at each station. Periods with missing entries indicate intervals during which no valid data were available. Overall, quantitative precipitation measurements showed good inter-station agreement.

(b) Correlation analysis of precipitation-related variables between Bochum and the neighboring stations, quantified using Pearson’s R. Correlation coefficients were lower for the categorical precipitation type than for quantitative measures (precipitation amount and snow depth). Each data point represents a single daily measurement.

S4 Fig.

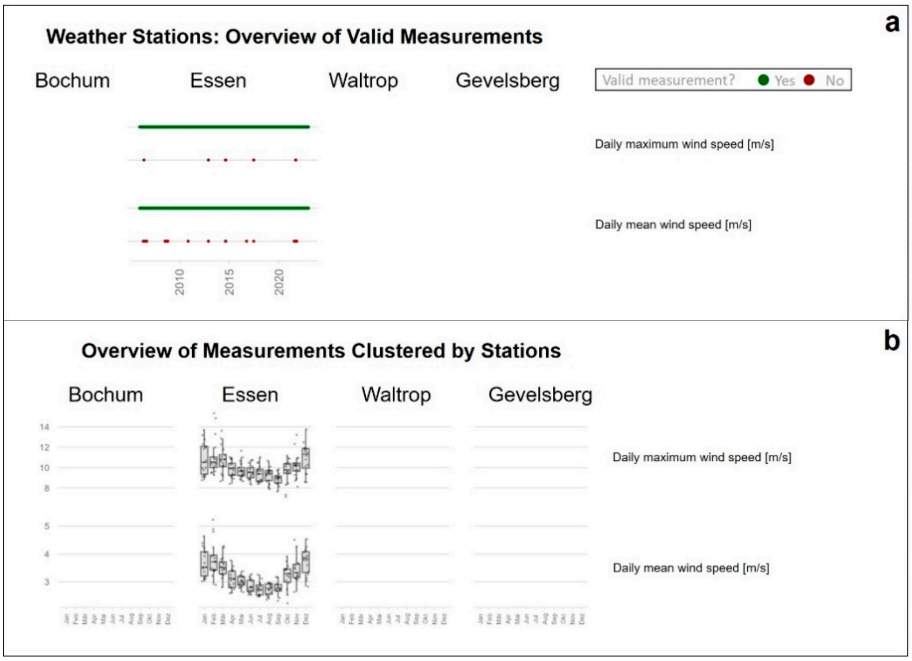

S4 Fig. Cluster C: Wind conditions.

(a) Temporal availability of wind-related measurements across the included meteorological stations. Wind speed data (daily maximum and daily mean) were available exclusively from the Essen-Bredeney station, while corresponding measurements were not recorded at the other stations during the observation period.

(b) Distribution of wind speed measurements recorded at the Essen station. The observed values showed plausible temporal patterns and were therefore used for subsequent analyses. Each data point represents the annual arithmetic mean of daily measurements for a given calendar year (2005–2022).

S5 Fig.

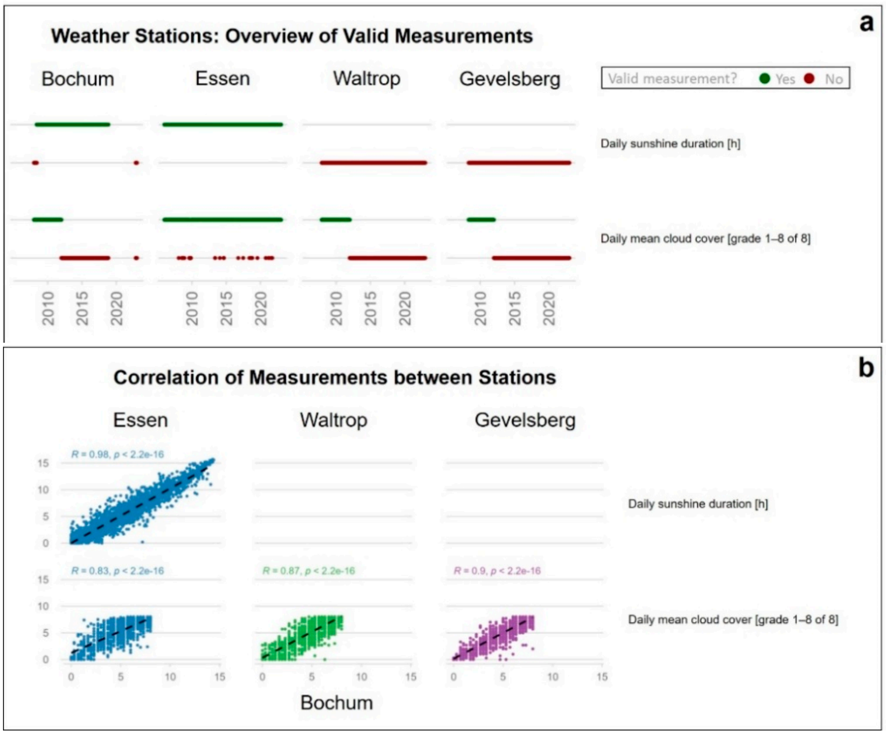

S5 Fig. Cluster D: Sunshine duration and cloud cover.
Daily measurement series for sunshine duration (h) and mean cloud cover (grades 1–8 of 8).

(a) Temporal availability of sunshine and cloud cover measurements across the included meteorological stations. In addition to known time gaps, extended periods of invalid measurements were observed for cloud cover at the Bochum station.

(b) Correlation analysis of sunshine duration and cloud cover measurements between Bochum and the neighboring stations, quantified using Pearson's R, demonstrated strong inter-station agreement for both parameters. Each data point represents a single daily measurement.

S6 Fig.

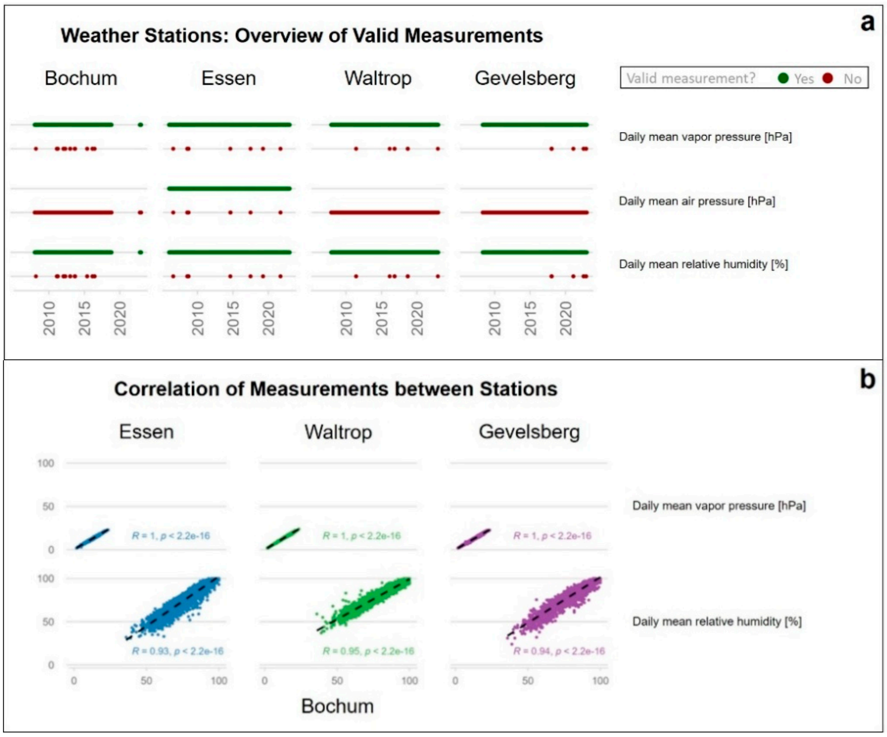

S6 Fig. Cluster E: Humidity and air pressure.

Measurement series for mean vapor pressure (hPa), mean air pressure (hPa), and mean relative humidity (%).

(a) Temporal availability of humidity- and pressure-related measurements across the included meteorological stations. No valid air pressure measurements were available for the Bochum station; corresponding data were therefore supplemented using measurements from the neighboring Essen station.

(b) Correlation analysis of atmospheric measurements between Bochum and the neighboring stations, quantified using Pearson's R, demonstrated strong inter-station agreement for basic atmospheric parameters. Each data point represents a single daily measurement.

Table S1. Complementary three-class ordinal benchmark analysis for low-, elevated-, and high risk weeks on the held-out test split.

Model	Accuracy	Macro recall	Macro F1	Weighted F1	Recall low	Recall elevated	Recall high
MLP	0.760	0.645	0.628	0.761	0.847	0.487	0.600
Weather-enhanced multinomial logistic regression	0.691	0.708	0.583	0.720	0.710	0.615	0.800
Indicator-enhanced multinomial logistic regression	0.669	0.704	0.558	0.703	0.672	0.641	0.800
Shallow decision tree	0.651	0.679	0.525	0.689	0.672	0.564	0.800
Calendar-only multinomial logistic regression	0.629	0.662	0.486	0.677	0.649	0.538	0.800
Always low risk	0.749	0.333	0.285	0.641	1.000	0.000	0.000

Note: Three ordinal risk categories were evaluated: low-, elevated-, and high-risk weeks. Macro F1 gives equal weight to each class and is therefore informative under class imbalance.
